# Multispectral imaging for MicroChip electrophoresis enables point-of-care newborn hemoglobin variant screening

**DOI:** 10.1016/j.heliyon.2022.e11778

**Published:** 2022-11-28

**Authors:** Ran An, Yuning Huang, Anne Rocheleau, Alireza Avanaki, Priyaleela Thota, Qiaochu Zhang, Yuncheng Man, Zoe Sekyonda, Catherine I. Segbefia, Yvonne Dei-Adomakoh, Enoch Mensah, Kwaku Ohene-Frempong, Isaac Odame, Amma Owusu-Ansah, Umut A. Gurkan

**Affiliations:** a Department of Biomedical Engineering, University of Houston, Houston, Texas, USA; b Department of Biomedical Sciences, University of Houston, Houston, TX, USA; c Department of Mechanical and Aerospace Engineering, Case Western Reserve University, Cleveland, OH, USA; d HemexHealth, Inc, Portland, OR, USA; e Department of Biomedical Engineering, Case Western Reserve University, Cleveland, OH, USA; f Department of Child Health, University of Ghana Medical School, Accra, Ghana; g Korle Bu Teaching Hospital, Accra, Ghana; h Department of Hematology, University of Ghana Medical School, Accra, Ghana; i Sickle Cell Foundation of Ghana, Kumasi, Ghana; j Division of Hematology/Oncology, The Hospital for Sick Children, Toronto, Ontario, Canada; k Department of Pediatrics, University of Toronto, Toronto, Ontario, Canada; l Department of Pediatrics, Division of Hematology and Oncology, University Hospitals Rainbow Babies and Children’s Hospital, Case Western Reserve University, Cleveland, OH, USA; m Case Comprehensive Cancer Center, Case Western Reserve University, Cleveland, OH, USA

**Keywords:** Newborn screening, Genetic hemoglobin disorders, Sickle cell disease, Multispectral imaging, Point-of-care diagnostics

## Abstract

Hemoglobin (Hb) disorders affect nearly 7% of the world’s population. Globally, around 400,000 babies are born annually with sickle cell disease (SCD), primarily in sub-Saharan Africa where morbidity and mortality rates are high. Screening, early diagnosis, and monitoring are not widely accessible due to technical challenges and cost. We hypothesized that multispectral imaging will allow sensitive hemoglobin variant identification in existing affordable paper-based Hb electrophoresis. To test this hypothesis, we developed the first integrated point-of-care multispectral Hb variant test: Gazelle-Multispectral. Here, we evaluated the accuracy of Gazelle-Multispectral for Hb variant newborn screening in 265 newborns with known hemoglobin variants including hemoglobin A (Hb A), hemoglobin F (Hb F), hemoglobin S (Hb S) and hemoglobin C (Hb C). Gazelle-Multispectral detected levels of Hb A, Hb F, Hb S, and Hb C/E/A_2_, demonstrated high correlations with the results reported by laboratory gold standard high performance liquid chromatography (HPLC) at Pearson Correlation Coefficient = 0.97, 0.97, 0.93, and 0.95. Gazelle-Multispectral demonstrated accuracy of 96.8% in subjects of 0–3 days, and 96.9% in newborns. The ability to obtain accurate results on newborn samples suggest that Gazelle-Multispectral can be suitable for large-scale newborn screening and for diagnosis of SCD in low resource settings.

## 1 Introduction

Hemoglobin (Hb) disorders including sickle cell disease (SCD) are among the world’s most common monogenic diseases [1]. Globally, an estimated 400,000 babies are born annually with SCD and 70%–75% are in sub-Saharan Africa (SSA) [2, 3, 4]. It is estimated that 50–90% of patients with SCD in SSA die by their 5th birthday [5, 6, 7]. However the World Health Organization (WHO) estimates that early diagnosis of SCD coupled with intervention programs would prevent 70% of existing SCD mortality [8].

Newborn infant, or neonate, is a child under 28 days of age defined by the WHO [9]. Effective management of SCD involves genetic counseling, early diagnosis through newborn screening and comprehensive care [10, 11, 12, 13]. SCD newborn screening performed in centralized laboratories has dramatically reduced SCD mortality in resource-rich countries [5, 14]. However, in sub-Saharan Africa and central India, where >90% of annual SCD births occur, newborn screening programs have not been implemented universally, if at all, due in large part to the cost and logistical burden of laboratory diagnostic tests [15].

SCD newborn screening requires sensitive detection of low levels of certain Hb variants in the context of high levels of expression of other Hb variants. For example, among newborns, normal hemoglobin A (Hb A) and sickle hemoglobin S (Hb S) are expressed at lower levels while fetal hemoglobin (Hb F) is highly expressed making up to 90% of total Hb [16]. The current centralized tests used for newborn screening of SCD are high performance liquid chromatography (HPLC) and isoelectric focusing (IEF). These tests rely on unaffordable (15k–35k US Dollar, or 90k–210k Ghanaian Cedi) specialized instruments, laboratory facilities, and highly trained personnel, which are lacking in low resource settings where SCD is most prevalent [17]. While the most up-to-date models of HPLCs allow automated sample processing, such advanced instruments are normally lacking in low resource settings. Additionally, conducting daily control tests, maintaining and troubleshooting for HPLCs [18] and HPLC autosamplers [19] still require trained personnel, which are also lacking in these settings. IEF is a less expensive central test option which can be, but is not usually used for quantification of Hb variants, misses certain Hb variants and requires skilled interpretation. Major hospitals in low-resource settings may have access to manual electrophoresis devices but processing samples with these devices are time consuming, need a laboratory setting, require expertise to read, therefore suffers from relatively slow turnaround of test results and start of treatment. Overall, these relatively advanced laboratory techniques require state-of-the-art facilities, which are lacking or in short supply in countries where the prevalence of hemoglobin disorders is the highest [20, 21]. As a result, there is a need for affordable, portable, easy-to-use, accurate, point-of-care (POC) tests to facilitate decentralized hemoglobin testing in low-resource settings to enable nationwide newborn screening.

Several POC diagnostic systems for SCD have been described [22, 23, 24] based on testing methods such as sickle cell solubility test and antibody-based lateral flow assays such as Sickle SCAN™ and HemoType SC™ [25, 26, 27]. However, the sickle cell solubility test is not reliable for samples with Hb S levels below 20% [25]. As a result, this method is not suited for screening for Hb S in newborns, where Hb S levels are normally below 20% [28, 29, 30]. Antibody-based lateral flow assays only report qualitative instead of quantitative test results [26]. Additionally, most of these tests only detect three hemoglobin variants (Hb A, Hb S, and Hb C) while missing Hb F and others, which may result in compromised detection sensitivity and specificity. Moreover, these tests lack readers embedded with data analysis software for result interpretation or electronic record keeping, and rely on subjective visual interpretation and manual recording of the test results, which are prone to errors in the field [31]. In fact, user misinterpretation and data entry errors have been reported to range between 2.3% to 26.9%, which can significantly compromise the accuracy of these tests [27].

**Figure 1:**
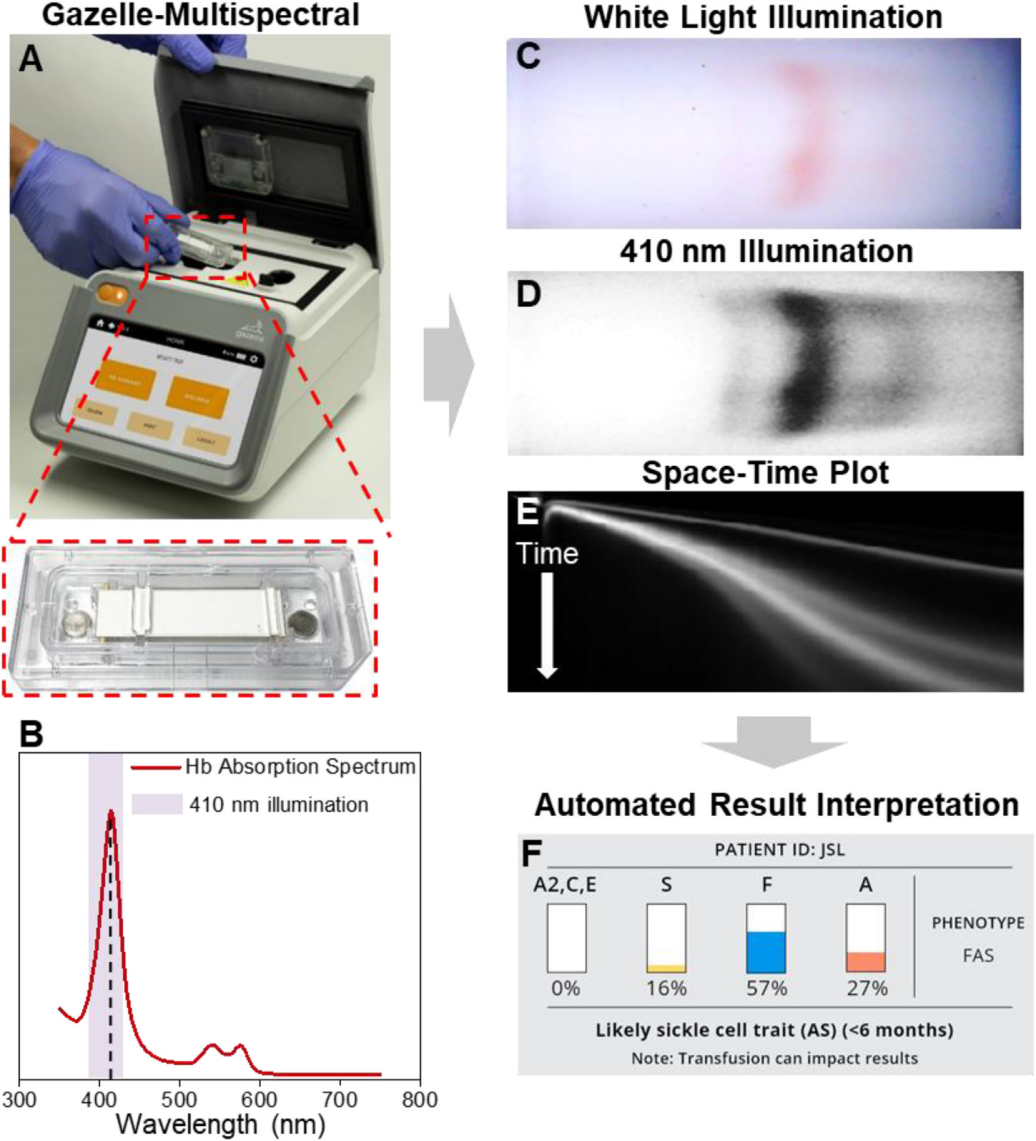
Gazelle-Multispectral for screening hemoglobin variants in newborns. (A) Gazelle-Multispectral platform for paper-based microchip electrophoresis using disposable cartridge (red box) at the point of need. (B) Hemoglobin absorption spectrum. Purple shaded area covers more than 90% of the power output according to the LED manufacturing specification and our internal testing. The Gazelle-Multispectral perform real time imaging and data analysis tracking the Hb electrophoresis process under both white light illumination (C) and 410 nm illumination (D). The images captured under white light illumination provides visual validation of test progression. (E) The space-time plots generated based on the images captures under 410 nm illumination are used for identifying and quantifying Hb variants in real time using an internally integrated data analysis algorithm. (F) At the end of each test, Gazelle-Multispectral algorithm automatically reports the identification and quantification of Hb variant results, and determines the patient phenotype accordingly.

In a 2019 report, the World Health Organization (WHO) listed hemoglobin testing as one of the most essential in vitro diagnostic (IVD) tests for primary care use in low and middle income countries [32]. Furthermore, hemoglobin electrophoresis has recently been added to the WHO essential list of IVDs for diagnosing SCD and sickle cell trait [33]. Leveraging the WHO recognized Hb electrophoresis test, we developed a paper-based, miniaturized Hb electrophoresis platform, Gazelle™ (Figure 1) [20,34,35]. Gazelle™ instrument is currently being sold from 1200 to 1500 USD (7212–9015 Ghanaian Cedi) to clinics and laboratories with the cost per test is in the range of 2–2.5 USD (12–15 Ghanaian Cedi) including all consumables. Gazelle has been tested in clinical studies in 4 different countries with more than 700 subjects, and demonstrated capability of identifying Hb variants with a limit of detection of 12.5% for Hb S [21, 35, 36]. As stated in our previous publication, however, Gazelle™ could not generate accurate results in subjects with high Hb F therefore was only for use on subjects of 6 weeks or older.

Here, we implemented multispectral imaging under both white illumination and 410 nm wavelength and developed the Gazelle-Multispectral platform as the first POC test able to identify and quantify Hb variants in newborns and people of any age. The high absorbance of hemoglobin at 410 nm wavelength [37] enhances the limit of detection thus allows detection and identification of hemoglobin variants at low concentrations, which is crucial for SCD screening in newborns. In this manuscript, we firstly determined the limit of detection of the Gazelle-Multispectral platform in laboratory environment using controlled samples in Portland, OR, USA. Additionally, we describe a study for evaluating the diagnostic performance of this platform for screening HbSS, HbSC disease, and the related carrier states (Hb S trait and Hb C trait) using whole blood at the POC in 441 subjects in Korle Bu, Ghana, a location selected for its high prevalence of both the Hb S and Hb C variants. Gazelle-Multispectral demonstrated overall accuracies of 96.8% in subjects of 0–3 days, 96.9% in newborns and 98.1% in all subjects, compared to HPLC. These results suggest that Gazelle-Multispectral is potentially suitable for large-scale newborn screening and diagnosis.

## 2 Methods

### 2.1 Laboratory determination of limit of detection

Gazelle Multispectral’s lower limit of detection for Hb S was determined using artificially created samples by mixing a cord blood sample with known Hb F level (12.9% Hb A, 82.8% Hb F, 0% Hb A_2_, determined by HPLC) and a blood sample from a patient with SCD undergoing hydroxyurea therapy with known levels of Hb A and Hb S (1.7% Hb A, 82.9% Hb S, CC% Hb F, 12.2% Hb F, 2.8% Hb A_2_, determined by HPLC). Hb S levels in the artificially created samples were obtained by HPLC as 11.0%, 7.9%, 7.1%, 4.2%, 3.3%, 2.2%, and 0.9%. Each artificially created sample was tested 3 times using Gazelle-Multispectral and the reported results were compared with Hb S levels determined by HPLC (Table S3). Further tests have been performed using additional 2 artificially created samples with Hb S levels at 3.3% and 3.7%. Each of these two additional samples was tested 10 times using Gazelle-Multispectral and the reported results were compared with Hb S levels determined by HPLC (Table S4).

### 2.2 Study design and oversight

We conducted a prospective diagnostic accuracy study on Gazelle-Multispectral for detecting Hb variants including Hb A, Hb F, Hb S, and Hb C at Korle Bu Teaching Hospital (KBTH), Accra, Ghana using an Institutional Review Board (IRB) approved protocol. The results obtained using the investigational assay, Gazelle-Multispectral, were compared to the results reported by the reference (“Gold-standard”) tests using HPLC. The Gazelle platform was designed and provided by Hemex Health, headquartered in Portland, Oregon, USA. The laboratory standard test used in the study was HPLC at KBTH. All authors have reviewed and analyzed the data and attest to their accuracy and completeness as well the fidelity of adherence to the study protocol.

### 2.3 Study populations and procedures

This test was conducted at KBTH, the largest public hospital in Ghana. Newborns were enrolled from the postnatal wards, and children were enrolled from the Child Welfare Clinic, during routine immunization visits. All newborns from the postnatal wards and the immunization clinic with parents' consent were tested. Subjects were excluded only if there had been a blood transfusion in the preceding 3 months. To our best knowledge, none of the children had an existing diagnosis of SCD. The study was approved by the KBTH IRB and informed consent obtained from each participant’s parent or guardian. A blood sample was obtained from each participant using finger prick at the vaccination clinic or heel prick for newborns. Any blood samples not tested immediately on Gazelle-Multispectral were refrigerated until use. Multiple local laboratory technicians performed the tests. The users had basic laboratory skills such as pipetting and vortexing and were able to independently perform the tests with less than 2 h training. After a Gazelle test was conducted, the remaining blood from the blood collection tube was saved and frozen at −80 °C. HPLC tests were performed at KBTH on the frozen samples using the D-10 HPLC system (Bio-Rad Laboratories, Hercules, CA, USA).

### 2.4 Gazelle multispectral test procedure

The technicians performed the tests according to the Gazelle-Multispectral instructions for use as published previously [35]. Briefly, 20 μl of blood and 40 μl of Gazelle Marker Fluid was pipetted into an Eppendorf tube which was then vortexed for 20 s to lyse the blood. 50 μl of Gazelle Buffer was used to wet the Gazelle Hb Variant Cartridge paper and the cartridge was soaked for 1 min. 20 μl of the blood mixture was pipetted onto a glass slide and a customized stamper was touched to the mixture. The blood sample was wicked and filled the stamper completely. The stamper stand was placed directly over the cartridge and the stamper with blood and marker was placed into the stamper stand. The operator held down the stamper stand for 5 s to apply the blood and marker mixture to the cartridge. The cartridge was flipped over and 200 μl Gazelle Buffer were pipetted into each of the wells on each end of the cartridge. Finally, the cartridge was placed into the Gazelle Reader and the test was started. After 8 min, the results screen showed the percentages of each hemoglobin type present in the blood sample as well as an interpretative statement. Each Gazelle-Multispectral test was completed within a total of 10 min including sample preparation and testing.

### 2.5 Confirmatory laboratory procedures

Blood samples that were stored at −80 °C were retrieved and thawed. 5 μl of sample was pipetted and diluted with 1500 μl of distilled water. Diluted hemolysates were arranged on racks and loaded into the Bio-Rad D-10 HPLC system. Each sample was tested for approximately 6 min. The results reported for each blood sample included the relative percentages of each hemoglobin type present.

### 2.6 Gazelle-Multispectral data analysis

Customized data analysis algorithm was integrated in Gazelle-Multispectral system. This data analysis algorithm automatically identifies sickle cell disease (FS, FSC, FSA), sickle cell trait (FAS), Hemoglobin C Trait (FAC), and normal phenotype (FA) based on Hb band migration pattern as described previously [35]. The data analysis algorithm also automatically quantifies the relative percentages of Hb A, Hb F, Hb S, and combined Hb C/E/A_2_. In this particular study, and all Hb variant recognized as Hb C/E/A_2_ were identified as Hb C due to the test location. The Gazelle-Multispectral reported Hb variant identification and quantification results were compared with the ones reported by HPLC using Pearson correlation and Bland-Altman analysis. Gazelle-Multispectral sensitivity, specificity, positive predictive value (PPV), and negative predictive value (NPV) in identification of SCD (FS/FSC/FSA) vs. Normal (FA), SCD (FS/FSC/FSA) vs. Sickle Cell Trait (FAS) and Hemoglobin C Trait (FAC), and Sickle Cell Trait (FAS) and Hemoglobin C Trait (FAC) vs. Normal (FA) were calculated for the study population compare to HPLC reported results.

The primary objective was to determine the limit of detection for detecting individual Hb variants, as well as the sensitivity, specificity, PPV, and NPV, of Gazelle Multispectral, compared to reference tests, in detecting normal Hb (Hb A), fetal Hb (Hb F), and common pathologic Hb variants (Hb S and Hb C), in whole blood specimens from newborns and older children. The main goal was to test the ability of Gazelle Multispectral to accurately detect HbSS, HbSC disease, and the related carrier states (Hb S trait and Hb C trait) in newborns and older children.

## 3 Results

### 3.1 Test population

A total of 441 subjects were tested using both Gazelle-Multispectral and HPLC acquired at KBTH, Ghana were included in this study. In this study, 265 out of 441 subjects were newborns within 28 days old. 250 out of the 265 newborns were 0–3 days old from the maternity ward before they left the hospital following birth and includes over 50% of the entire test population (250 out of 441 subjects). Given the clinical importance of newborns from 0 to 3 days old, we report the test results separately for this group of subjects (250 subjects) and for newborns from 4 to 28 days old (15 subjects). Additionally, Hb F levels are known to decrease at a rate of ∼5% per week till normally becoming negligible at 6 months [38]. As a result, we also report, in supplementary information, 176 out of 441 test results for infants from 28 days to 6 months tested at the vaccination clinic, within whom the Hb F levels remain high. A more detailed summary for the age of the subjects is included in the supplementary information (Table S1).

### 3.2 Gazelle-Multispectral result reporting

Gazelle-Multispectral algorithm verifies the quality of test results according to the internally embedded data quality control (QC) method. According to the QC method, Gazelle-Multispectral organizes test results under one of the 3 categories: 1) ‘Valid’ test; 2) ‘Inconclusive’ test without interpretation; and 3) ‘Inconclusive’ test with a possible interpretation. The ‘Valid’ and ‘Inconclusive’ tests were defined according to published recommendations in the literature [39] and the STARD guidelines [40]. A ‘Valid’ test was defined as a test that performed as expected according to objective standards and the test result was reported properly from the data analysis algorithm. An ‘Inconclusive’ test was a test that performed adequately according to an objective set of standards. However, an ‘Inconclusive’ test has quantification confidence value automatically evaluated by the algorithm that is lower than the preset threshold value, which can be recognized at the end of the test. Reasons for ‘Inconclusive’ tests include appearance of a band or bands at or close to the borderline region between two adjacent detection windows.

In this study, test results from 216 out of 250 (86.4%), 11 out of 15 (73.3%), and 138 out of 176 (78.4%) subjects were categorized as ‘Valid’ for subjects within 0–3 days, 4–28 days, and 28 days–6 months, respectively (365) ‘Valid’ (82.8%) and 76 ‘Inconclusive’ (17.2%) tests out of 441 total tests. More detailed information is included in supplementary information (Table S2).

### 3.3 Gazelle-Multispectral separates, images, and tracks hemoglobin variants real-time under multi-spectrum during electrophoresis

The fundamental principle behind Gazelle-Multispectral technology is hemoglobin electrophoresis, in which different (bio)molecules including total hemoglobin, standard calibrator, and hemoglobin variants can be separated based on their charge-to-mass ratio when exposed to an electric field in the presence of a carrier substrate. Gazelle-Multispectral cartridge is single-use and can be mass-produced at low-cost (Figure 1A) [35]. Gazelle-Multispectral reader implemented imaging capture system under both white light field and at 410 nm wavelength. The 410 nm is selected to match the hemoglobin peak absorption wavelength and to increase limit of detection (Figure 1B). Tris/Borate/EDTA (TBE) buffer is used to provide the necessary ions for electrical conductivity at pH of 8.4 in the cellulose acetate paper. Hb molecules carry net negative charges under this pH and caused them to travel from the cathode to the anode upon exposure to electric field. The electric mobility differences of various hemoglobin phenotypes allow separation and thus identification of each hemoglobin variant. Separated hemoglobin variants are imaged under both white light illumination (Figure 1C) and 410 nm illumination (Figure 1D). The acquired data under white light illumination demonstrates the natural red color of hemoglobin and thus validates the Gazelle-Multispectral tests (Figure 1C). The acquired data under 410 nm illumination yields an enhanced limit of detection and a higher signal to background ratio than white light illumination data, and is used to construct the space-time plot demonstrating the entire process of electrophoresis. The Gazelle-Multispectral algorithm then utilizes the space-time plot for sensitive and accurate identification and quantification of Hb variants (Figure 1E and F). Combining both white light and 410 nm detection spectrums, Gazelle-Multispectral automatically tracks, detects, identifies and quantifies electrophoretically separated low concentration hemoglobin variants within the 8-minutes test time using pre-embedded algorithm and report test results at the moment of test completion.

### 3.4 Analysis of limit of detection

In the analytical assessment, the limit of detection (LOD) was determined as the lowest concentration for which all three replicates scored positive. Gazelle-Multispectral consistently identified Hb S in 3 out of 3 replicates for artificially created samples with Hb S levels at 11.0%, 7.9%, 7.1%, 4.2%, and 3.3%. However, Gazelle-Multispectral identified Hb S in 2 out of 3 replicates for artificially created samples with Hb S levels at 2.2% and 0 out of 3 for artificially created samples with Hb S levels at 0.9% (Table. S3). 20 additional confirmatory tests were performed using 2 additional artificially created samples with Hb S levels at 3.3% and 3.7% (determined by HPLC). Gazelle-Multispectral consistently identified Hb S in 10 out of 10 replicates in these tests for both samples (total of 20 tests). As a result, the lower LOD of Gazelle-Multispectral for identifying Hb S was set at 4%. This 4% lower LOD is sufficient for detecting the low percentage of Hb S typically found in newborn samples for newborns with sickle cell trait (FAS: Hb S 6.5 ± 2.8%) and SCD (FS: Hb S 10.2 ± 3.9%) [41]. Figure S2 demonstrates a representative test result for sample with HbS around Gazelle-Multispectral limit of detection, where the test result for Gazelle-Multispectral vs. HPLC was: HbF = 87% vs. 85%, HbA = 9% vs. 10%, and HbS = 4% vs. 5%.

### 3.5 Gazelle-Multispectral automatically validates electrophoresis separation, identifies low concentration hemoglobin, and determines their relative percentages based on multi-spectrum imaging

**Figure 2:**
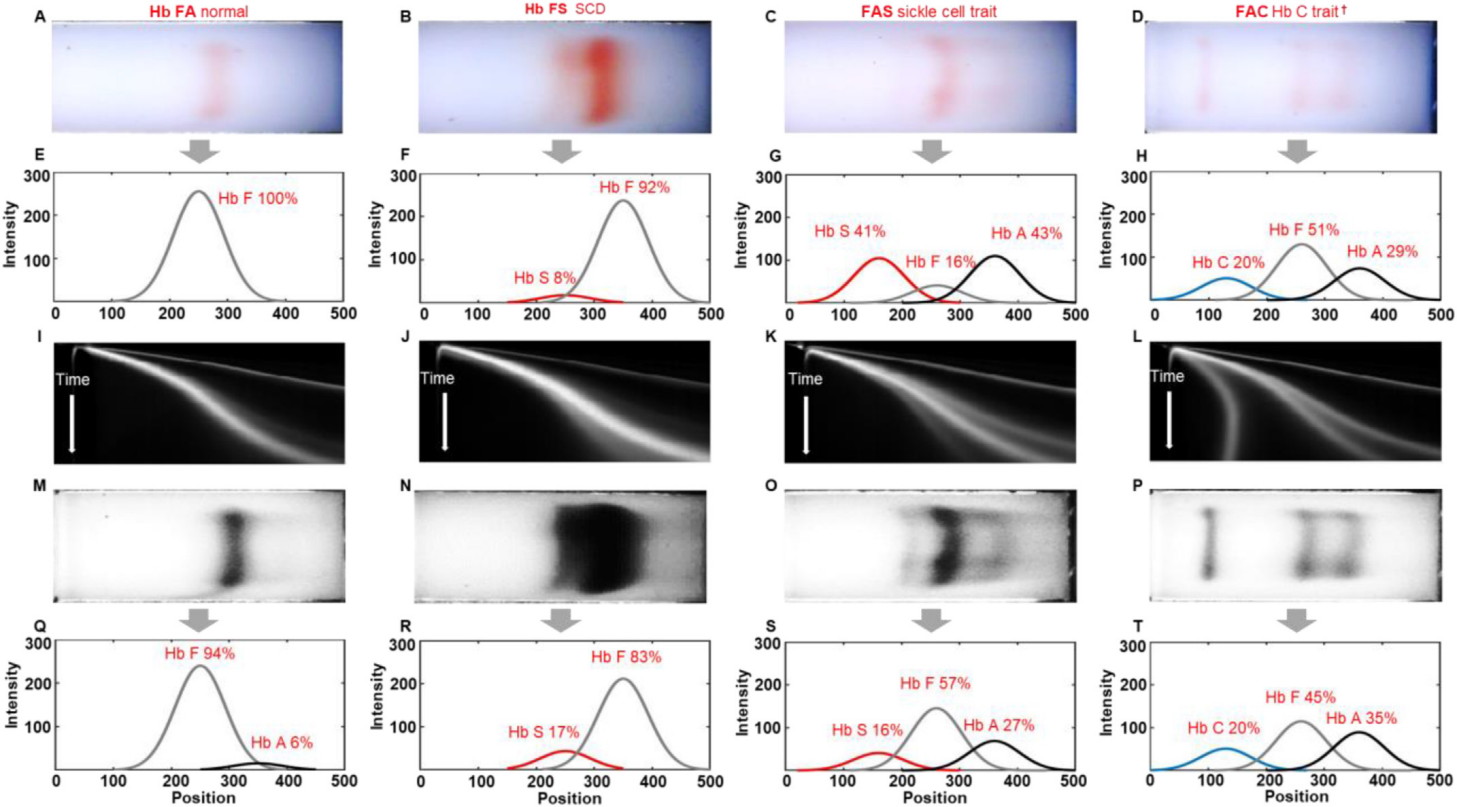
Identification of Hb variants and quantification of Hb percentages by Gazelle-Multispectral. (A-D) The first row shows images captured under white light field. (E-H) The second row shows electropherograms reconstructed by the white data analysis algorithm based on the white light images (electropherograms not visible by users in the field). (I-L) The third row illustrates 2D representation of Gazelle-Multispectral space-time plots of band migration in 410 nm imaging mode, which are used with the machine learning algorithm for identifying Hb variants. (M-P) The fourth row shows images captured under 410 nm wavelength. (Q-T) The fifth row shows electropherograms reconstructed by the data analysis algorithm based on the 410 nm images captured at the same time as the white light images. The Gazelle-Multispectral data analysis algorithm sensitively and accurately identified Hb variants agreeing with HPLC reported results. Column 1–4: Multispectral test results for samples with different phenotypes. Column 1: Hb FA (Healthy newborn, Hb A: 8% vs. 6%, Hb F: 92% vs. 94%, Gazelle-Multispectral vs. HPLC); Column 2: Hb FS (Newborn with sickle cell disease, Hb F 83% vs. 89%, Hb S 17% vs. 11%, Gazelle-Multispectral vs. HPLC); Column 3: Hb FAS (Newborn with sickle cell trait, Hb S 16% vs. 16%, Hb F 57% vs. 55%, Hb A 27% vs. 29%, Gazelle-Multispectral vs. HPLC); and Column 4: Hb FAC (Newborn with Hb C disease, Hb C 20% vs. 20%, Hb F 45% vs. 45%, Hb A 35% vs. 35%, Gazelle-Multispectral vs. HPLC). Gazelle-Multispectral enabled identification and quantification of low concentration Hb variants with higher sensitivity (I–T) compared to white light imaging mode (A-H). †: Gazelle-Multispectral reports Hb C/E/A_2_ as demonstrated in Figure 1E. The identified Hb C/E/A_2_ were recognized as Hb C according to test location (Ghana).

Four representative tests with different Hb variants were demonstrated in Figure 2 (**Column 1**: Healthy newborn, FA; **Column 2**: Newborn with SCD, FS; **Column 3**: Newborn with Sickle Cell Trait, FAS; and **Column 4**: Newborn Hemoglobin C Trait, FAC). For each test, Gazelle-Multispectral algorithm recognizes the initial application point of the mixture containing a blue control marker and hemoglobin. The algorithm then distinguishes and tracks the blue marker and hemoglobin according to their naturally distinct blue and red colors within the white light field during separation. The tracked blue marker and red hemoglobin migration pattern is analyzed by the image processing and decision algorithm to determine test validity (Figure 2A–H). A validated test is further analyzed utilizing data acquired under 410 nm (Figure 2I–T). A space-time plot is generated to illustrate the entire band migration on the paper (x-axis, from left to right) within the entire time (y-axis, from top to bottom, Figure 2I–L). Tests validated under white light field are further analyzed utilizing the space-time plots acquired under 410 nm (Figure 2I–L).

Electropherograms were regenerated based on single images acquired under white light field (Figure 2E–H) and 410 nm wavelength (Figure 2Q–T). These electropherograms are only used to compare the Hb variant identification and quantification capabilities between images captured under white light field and 410 nm wavelength specifically in this manuscript, and are not used for data interpretation during field test. The original Hb bands information captured under 410 nm wavelength are available in Figure S1. These band information were used to generate the electropherograms shown in Figure 2(Q–T). As stated in our previous publication [35], in samples with high Hb F levels, Hb variants identified and quantified under white light field deviated from the ones reported by HPLC therefore only served to validate the tests in the new Gazelle-Multispectral tests. In comparison, Gazelle-Multispectral reported in this manuscript sensitively and accurately identified Hb variants agreeing with HPLC reported results in all these 4 representative samples (Gazelle-Multispectral vs. HPLC; FA: Hb F: 92% vs. 94%; Hb A: 8% vs. 6%; FS: Hb F 83% vs. 89%, Hb S 17% vs. 11%; FAS: Hb F 57% vs. 55%, Hb A 27% vs. 29%, Hb S 16% vs. 16%; and FAC: Hb F 45% vs. 45%, Hb A 35% vs. 35%, Hb C 20% vs. 20%).

### 3.6 Gazelle-Multispectral Hb variant quantification demonstrated high correlation with HPLC

**Figure 3:**
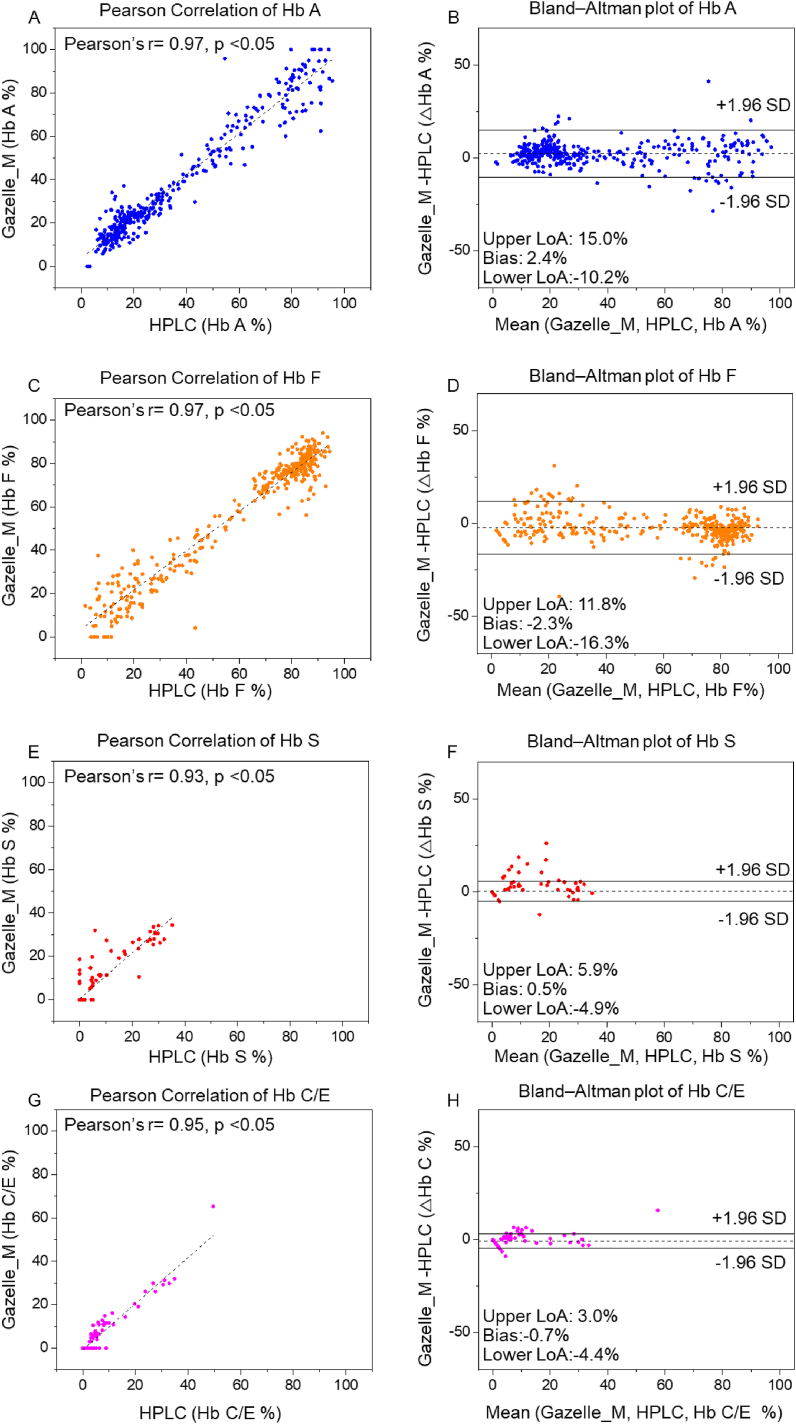
Gazelle-Multispectral Hb variant identification and quantification in all test subjects. Pearson correlation (Column 1) and Bland-Altman analysis (Column 2) showed Gazelle-Multispectral identified and quantified Hb A (A&B, Pearson coefficient correlation (PCC) = 0.97, p < 0.05, Mean bias ±1.96 × Standard Deviation (SD) = 2.4% ± 12.6%), Hb F (C&D, PCC = 0.97, p < 0.05, Mean bias ±1.96SD = −2.3% ± −14.0%), Hb S (E&F, PCC = 0.93, p < 0.05, Mean bias ±1.96 SD = 0.5% ± 5.4%), and Hb C levels (G&H, PCC = 0.95, p < 0.05, Mean bias ±1.96SD = −0.7% ± 3.3%) agree with the ones reported by laboratory standard HPLC. In Column 2, the solid black lines indicate the mean biases and the dashed gray lines represent 95% limits of agreement. **∗:** 365 ‘Valid’ tests out of 441 total tests were included in this correlation calculation. ‘Inconclusive’ tests did not generate a result that could be included in the correlation coefficient calculation [39, 40].

Pearson correlation analysis and Bland-Altman analysis were performed on the 365 tests recognized as ‘Valid’ (including 216 valid tests from subjects of 0–3 days, 11 valid tests from subjects of 4–28 days, and 138 valid tests valid tests from subjects of 28 days–6 months (Table S2)). The correlation plots include the Gazelle-Multispectral determined Hb variant levels (y axis) versus the Hb variant levels reported by the HPLC (x axis) including Hb A, Hb F, Hb S, and Hb C (Figure 3A, C, E, G). The Bland-Altman analysis plots demonstrate the difference between Gazelle-Multispectral determined Hb variant levels (y axis) at the entire range of Hb levels detected (x axis, Figure 3B, D, F, H). The results from Pearson correlation analysis demonstrate Pearson correlation coefficient (PCC) of 0.97, 0.97, 0.93, and 0.95 for Hb A, Hb F, Hb S, and Hb C. Bland-Altman analysis showed Gazelle-Multispectral determines blood Hb variant levels with mean bias of 2.4% for Hb A (Limits of agreement, LOA: −10.2%–15.0%); −2.3% for Hb F (LOA: −16.3%–11.8%); 0. 5% for Hb S (LOA: −4.9%–5.9%), and −0.7% for Hb C (LOA: −4.4%–3.0%, Figure 3, Second Column). Together, these results revealed acceptable agreement between Gazelle-Multispectral determined Hb variant levels and HPLC reported Hb variant levels (Figure 3, First Column).

### 3.7 Sensitivity and specificity of Gazelle-Multispectral SCD newborn screening

In this clinical study, Gazelle-Multispectral test results included the following (Table 1 for newborns and Table S5 for subjects from 28 days to 6 months): disease (FS/FSC/FSA), Hb S (FAS), Hb C Trait (FAC), and normal (FA). Gazelle-Multispectral identified newborn subjects (<28 days) with disease including FS, FSC, and FSA from normal subjects and subjects with the related carrier states (Hb S trait, FAS and Hb C trait, FAC) with 100% sensitivity, specificity, PPV and NPV (Table 1 and Table S5) 6 subjects with normal Hb (FA) were identified as Sickle Cell Trait (FAS Table 1). 1 subject with Sickle Cell Trait (FAS) was identified as normal Hb (FA, Table 1). Sensitivity, specificity, PPV, and NPV for identifying subjects with the related carrier states from normal subjects (Trait vs. Normal) were 97.3%, 96.6%, 85.7%, and 99.4%. In tests conducted among subjects from 28 days to 6 months, Gazelle-Multispectral identified disease vs. normal, disease vs. trait, and trait vs. normal at 100% sensitivity, specificity, PPV, and NPV. As a result, Gazelle-multispectral demonstrated an accuracy of 96.8% in subjects of 0–3 days (209 correct in 216 valid tests), and accuracy of 96.9% (220 correct in 227 valid tests) in newborns and an overall accuracy of 98.1% (358 correct in 365 valid tests) from the total 441 tests in all subjects, comparing to HPLC.

**Table 1:** Gazelle-Multispectral screening sensitivity, specificity, positive predictive value (PPV), and negative predictive value (NPV) in comparison to reference standard method^a^.

	Disease vs. Normal^b^		Disease vs. Trait^c^		Trait vs. Normal^d^	
	0–3 Days	4–28 Days	0–3 Days	4–28 Days	0–3 Days	4–28 Days
**True positive**, TP	2	0	2	0	36	1
**True negative**, TN	170	10	36	1	170	10
**False Positive**, FP	0	0	0	0	6	0
**False negative**, FN	0	0	0	0	1	0
**Sensitivity**, TP/(TP + FN)	100.0%	-	100.0%	-	97.3%	100%
**Specificity**, TN/(TN + FP)	100.0%	100%	100.0%	100%	96.6%	100%
**PPV**, TP/(TP + FP)	100.0%	-	100.0%	-	85.7%	100%
**NPV**, TN/(TN + FN)	100.0%	100%	100.0%	100%	99.4%	100%

a 216 and 11 ‘Valid’ tests out of 250 and 15 total tests for 0–3 days and 4–28 days subjects were included in this calculation. ‘Inconclusive’ tests did not generate a result that could be included in the sensitivity-specificity analysis [39, 40].

b SS/SC/FS/FSC vs. AA/FA.

c SS/SC/FS/FSC vs. AS/AC/FAS/FAC.

d AS/AC/FAS/FAC vs. AA/FA, 6 subjects with Hb FA were recognized as Hb FAS, 1 subject with Hb FAS was recognized as Hb FA.

## 4 Discussion

Hb F expression represents up to 90% of total Hb in newborns [28, 29]. The WHO estimated that early detection of SCD coupled with intervention programs would prevent 70% of existing SCD mortality [8]. Quantification of Hb variants percentage, while not critical in the newborn period, is nevertheless of clinical significance in determining follow up testing in certain circumstances. For instance, quantitative Hb variants detection can facilitate to distinguish sickle cell trait (FAS) from a S-beta+ Thalassemia (FSA) as the difference is the relative percentages of Hb A and Hb S. Without accurate quantification some S-beta+ thal infants could be called a sickle trait. Our previous system, Gazelle™, detects Hb variants only under white light. As a result, Gazelle™ demonstrated limit of detection at 12.5% for Hb S, and was not accurate for samples with high Hb F levels (Figure 2C and G), therefore was only for use on subjects of 6 weeks or older. Gazelle-Multispectral presented in this manuscript implements multispectral imaging into a point-of-care hemoglobin electrophoresis test, which allows sensitive detection and quantification of low concentration Hb S with a limit of detection at 4%, thus enables SCD screening for Hb variants among newborns having low levels of Hb A and Hb S. The Gazelle-Multispectral algorithm automatically provides Hb variant identification and quantification results of relative Hb percentages (Figure 1E) and does not require users to perform result interpretation. Additionally, Gazelle-Multispectral uses a low-cost disposable cartridge, requires only limited laboratory resources, be operated on battery power, and enables automated analysis and interpreted results along with digital secured storage of test results, which can reduce human errors and improves accessibility of public health data. These critical features distinguish Gazelle-Multispectral from current available laboratory methods and other emerging POC technologies and assist health-care professionals in diagnosing and screening patients with hemoglobin variants with affordable and simple, tests.

In this clinical study conducted among 441 subjects including 250 subjects from 0 to 3 days old, 15 subjects from 4 to 28 days old, and 176 subjects from 28 days to 6 months old in Korle Bu, Ghana, Gazelle-Multispectral demonstrated 100% sensitivity and specificity for identifying newborns with diseases vs. healthy subjects; and subjects with disease vs. subjects with sickle cell trait and Hb C trait; Additionally, Gazelle-Multispectral demonstrated 97.3% sensitivity and 96.6% specificity for identifying subjects with sickle cell trait and Hb C trait vs. healthy subjects. Common practice in Hb testing is that all positive test results are confirmed with a secondary method prior to final diagnostic decision making and treatment initiation [42]. Therefore, all disease positive tests would likely result in a secondary confirmatory test that should eliminate the small number of false positives.

Hb electrophoresis techniques, including this reported technology and standard laboratory tests such as capillary electrophoresis and IEF, overall share a common limitation in discriminating certain Hb variants due to their similar electrophoretic mobilities at given condition. For example, it is challenging to discern Hb E and Hb A_2_, as well as to discern rarer variants such as Hb D Punjab, Hb D Iran, Hb Lepore, and Hb Q India, using both Gazelle and laboratory capillary electrophoresis because these hemoglobin variants demonstrate partially overlapped peak within the same detection window [43, 44]. Peak overlapping (i.e., Hb G and Hb D) is also a challenge for the reference standard HPLC as well as its alternatives [43, 45, 46]. Notably, Hb C and Hb E, and Hb A_2_ are known to co-migrate in paper-based hemoglobin electrophoresis. Therefore, Gazelle-Multispectral reports Hb C/E/A_2_ instead of reporting Hb C or E or A_2_ individually (Figure 1E). However, these Hb variants display distinct geographical prevalence and distribution. For example, Hb C is highly prevalent in West Africa thus is related to this study [47], while Hb E is the most prevalent in the Mediterranean region, Southeast Asia, and in the Indian subcontinent [48, 49, 50, 51, 52]. As a result, test location, the ethnicity of the subject, and clinical history can be used to facilitate differentiate between these co-migrating Hb types. For example, in this particular study, and all Hb variant recognized as Hb C/E/A_2_ were identified as Hb C due to the test location. Overall, it is recommended with HPLC, CE, and Gazelle, that positive tests have a second confirmatory test using a different method.

In summary, Gazelle-Multispectral enables affordable and simple identification of common Hb variants in newborns at the point-of-need. The Gazelle-Multispectral reader provide animated on-screen instructions of step-by-step guidance for test operation procedures to minimize user errors. The internally integrated data analysis algorithm automatically reports Hb variant identification and quantification results in an objective and easily understandable manner. Gazelle-Multispectral is a versatile, mass-producible, multispectral detection-based electrophoresis platform technology for affordable and accurate diagnostic testing and newborn screening programs for SCD at the POC in low resource regions where the prevalence of SCD is high.

## Declarations

### Author contribution statement

RA, AR, AA, and UAG conceived and designed the experiments. RA, YH, and AA contributed to the proof-of-concept experiments and initial development. AR, PT, CS, YDA, EM, and IO helped with the planning and execution of clinical testing, including human subject research protocol development, subject recruitment, blood sample collection, and testing. RA and QZ performed the in house limit of detection tests and data analysis. RA, YH, AR, AA, QZ, YM, and ZS performed the data analysis, prepared the tables, figures, figure captions, and supplementary information. KOF and AOA assisted in data interpretation. RA drafted the manuscript and all authors edited the manuscript. RA, YH, AR, AA, PT, QZ, YM, ZS, CS, YDA, AOA, EM, KOF, IO, and UAG reviewed and edited the manuscript.

### Funding statement

Umut A. Gurkan was supported by 10.13039/100000050National Heart, Lung, and Blood Institute [R44HL140739; R41HL151015; R01HL133574], 10.13039/100000062National Institute of Diabetes and Digestive and Kidney Diseases [R41DK119048].

Sr. Research Associate Ran An was supported by 10.13039/100000050National Heart, Lung, and Blood Institute [1U54HL143541; T32HL134622; K25HL159358].

### Data availability statement

Data will be made available on request.

### Declaration of interest’s statement

The authors declare the following conflict of interests: RA, QZ, UAG, and Case Western Reserve University have financial interests in Hemex Health Inc. UAG and Case Western Reserve University have financial interests in BioChip Labs Inc. UAG and Case Western Reserve University have financial interests in Xatek Inc. UAG has financial interests in DxNow Inc. Financial interests include licensed intellectual property, stock ownership, research funding, employment, and consulting. Hemex Health Inc. offers point-of-care diagnostics for hemoglobin disorders, anemia, and malaria. BioChip Labs Inc. offers commercial clinical microfluidic biomarker assays for inherited or acquired blood disorder.

### Additional information

No additional information is available for this paper.

## Appendix A Supplementary data

Supplementary Information.Revision2 Supplementary Information.Revision2

## References

[1] The World Health Organization (2022). https://www.who.int/westernpacific/health-topics/newborn-health.

[2] Alapan Y., Fraiwan A., Kucukal E., Hasan M.N., Ung R., Kim M., Odame I., Little J.A., Gurkan U.A. (2016). Emerging point-of-care technologies for sickle cell disease screening and monitoring. Expet Rev. Med. Dev..

[3] McGann Patrick T., Hoppe Carolyn (2017). The pressing need for point-of-care diagnostics for sickle cell disease: a review of current and future technologies. Blood Cells, Mol. Dis..

[4] Hsu L., Nnodu O.E., Brown B.J., Tluway F., King S., Dogara L.G., Patil C., Shevkoplyas S.S., Lettre G., Cooper R.S., Gordeuk V.R., Tayo B.O. (2018). White paper: pathways to progress in newborn screening for sickle cell disease in sub-Saharan Africa. J. Trop. Dis. Publ. Health.

[5] El-Haj Nura, Hoppe Carolyn C. (2018). Newborn screening for SCD in the USA and Canada. Int. J. Neonatal Screen..

[6] Sabarense A.P., Lima G.O., Silva L.M., Viana M.B. (2015). Characterization of mortality in children with sickle cell disease diagnosed through the Newborn Screening Program. J. Pediatr.(Rio J).

[7] Wang Y., Liu G., Caggana M., Kennedy J., Zimmerman R., Oyeku S.O., Werner E.M., Grant A.M., Green N.S., Grosse S.D. (2015). Mortality of New York children with sickle cell disease identified through newborn screening. Genet. Med..

[8] Regional Committee for Africa (2011).

[9] The World Health Organization (2022). Newborn Health in the Western Pacific. https://www.who.int/westernpacific/health-topics/newborn-health.

[10] Quarmyne M.O., Dong W., Theodore R., Anand S., Barry V., Adisa O., Buchanan I.D., Bost J., Brown R.C., Joiner C.H., Lane P.A. (2017). Hydroxyurea effectiveness in children and adolescents with sickle cell anemia: a large retrospective, population-based cohort. Am. J. Hematol..

[11] Tshilolo Léon, George Tomlinson, Williams Thomas N., Santos Brígida, Olupot-Olupot Peter, Adam Lane, Aygun Banu, Stuber Susan E., Latham Teresa S., McGann Patrick T., Ware Russell E. (2018). Hydroxyurea for children with sickle cell anemia in sub-Saharan Africa. N. Engl. J. Med..

[12] Tubman V.N., Marshall R., Jallah W., Guo D., Ma C., Ohene-Frempong K., London W.B., Heeney M.M. (2016). Newborn screening for sickle cell disease in Liberia: a pilot study. Pediatr. Blood Cancer.

[13] Williams S.A., Browne-Ferdinand B., Smart Y., Morella K., Reed S.G., Kanter J. (2017). Newborn screening for sickle cell disease in St. Vincent and the grenadines: results of a pilot newborn screening program. Glob. Pediatr. Health.

[14] Therrell B.L., Lloyd-Puryear M.A., Eckman J.R., Mann M.Y. (2015). Newborn screening for sickle cell diseases in the United States: a review of data spanning 2 decades. Semin. Perinatol..

[15] Aygun Banu, Isaac Odame (2012). A global perspective on sickle cell disease. Pediatr. Blood Cancer.

[16] Allaf B., Patin F., Elion J., Couque N. (2018). New approach to accurate interpretation of sickle cell disease newborn screening by applying multiple of median cutoffs and ratios. Pediatr. Blood Cancer.

[17] Nagel R.L., Fabry M.E., Steinberg M.H. (2003). The paradox of hemoglobin SC disease. Blood Rev..

[18] Imad A., Haidar Ahmad (2017). Necessary Analytical Skills and Knowledge for Identifying, Understanding, and Performing HPLC Troubleshooting. Chromatographia.

[19] Paul Carsten, Frank Steiner, Dong Michael (2019).

[20] Ran An, Hasan Muhammad Noman, Man Yuncheng, Gurkan Umut A. (2019). Integrated anemia detection and hemoglobin variant identification using point-of-care microchip electrophoresis. Blood.

[21] Fraiwan Arwa, Hasan Muhammad Noman, Ran An, Xu Julia Z., Rezac Amy J., Kocmich Nicholas J., Oginni Tolulope, Olanipekun Grace Mfon, Hassan-Hanga Fatimah, Jibir Binta W., Gambo Safiya, Verma Anil K., Bharti Praveen K., Riolueang Suchada, Ngimhung Takdanai, Suksangpleng Thidarat, Thota Priyaleela, Shanmugam Rajasubramaniam, Das Aparup, Viprakasit Vip, Piccone Connie M., Little Jane A., Obaro Stephen K., Gurkan Umut A. (2019). International multi-site clinical validation of point-of-care microchip electrophoresis test for hemoglobin variant identification. Blood.

[22] Arishi W.A., Alhadrami H.A., Zourob M. (2021). Techniques for the detection of sickle cell disease: a review. Micromachines.

[23] Ilyas S., Simonson A.E., Asghar W. (2020). Emerging point-of-care technologies for sickle cell disease diagnostics. Clin. Chim. Acta.

[24] Jaja C., Edem-Hotah J., Shepherd J., Patel N., Xu H.Y., Gibson R.W. (2020). Analytic characteristics and performance of novel immunoassay point-of-care tests for early diagnosis of sickle cell disease A systematic review. Point Care.

[25] Canning D.M., Huntsman R.G. (1970). An assessment of Sickledex as an alternative to the sickling test. J. Clin. Pathol..

[26] Steele C., Sinski A., Asibey J., Hardy-Dessources M.D., Elana G., Brennan C., Odame I., Hoppe C., Geisberg M., Serrao E., Quinn C.T. (2019). Point-of-care screening for sickle cell disease in low-resource settings: a multi-center evaluation of HemoTypeSC, a novel rapid test. Am. J. Hematol..

[27] Kanter Julie, Telen Marilyn J., Hoppe Carolyn, Roberts Christopher L., Kim Jason S., Yang Xiaoxi (2015). Validation of a novel point of care testing device for sickle cell disease. BMC Med..

[28] Thomas Caroline, Lumb Andrew B. (2012). Physiology of haemoglobin. Cont. Educ. Anaesth. Crit. Care Pain.

[29] Metaxotou-Mavromati Anna D., Antonopoulou Helene K., Laskari Sophie S., Tsiarta Helene K., Ladis Vasilis A., Kattamis Christos A. (1982). Developmental changes in hemoglobin F levels during the first two years of life in normal and heterozygous β-Thalassemia infants. Pediatrics.

[30] Eastman J.W., Wong R., Liao C.L., Morales D.R. (1996). Automated HPLC screening of newborns for sickle cell anemia and other hemoglobinopathies. Clin. Chem..

[31] Ross L.F. (2010). Mandatory versus voluntary consent for newborn screening?. Kennedy Inst. Ethics J..

[32] Gevena: World Health Organization (2019).

[33] World Health Organization (WHO) (2019).

[34] An R., Hasan M.N., Man Y., Gurkan U.A. (2019).

[35] Hasan M.N., Fraiwan A., An R., Alapan Y., Ung R., Akkus A., Xu J.Z., Rezac A.J., Kocmich N.J., Creary M.S., Oginni T., Olanipekun G.M., Hassan-Hanga F., Jibir B.W., Gambo S., Verma A.K., Bharti P.K., Riolueang S., Ngimhung T., Suksangpleng T., Thota P., Werner G., Shanmugam R., Das A., Viprakasit V., Piccone C.M., Little J.A., Obaro S.K., Gurkan U.A. (2020). Paper-based microchip electrophoresis for point-of-care hemoglobin testing. Analyst.

[36] Kumar Ravindra, Mishra Sweta, Gwal Anil, Shanmugam Rajasubramaniam (2021). Evaluation of paper-based point of care screening test for sickle cell disease. Indian J. Clin. Biochem..

[37] Faber Dirk J., Mik Egbert G., Maurice C., Aalders G., van Leeuwen Ton G. (2003). Light absorption of (oxy-)hemoglobin assessed by spectroscopic optical coherence tomography. Opt Lett..

[38] Edoh Dominic, Antwi-Bosaiko Charles, Amuzu Dominic (2006). Fetal hemoglobin during infancy and in sickle cell adults. Afr. Health Sci..

[39] Shinkins Bethany, Thompson Matthew, Mallett Susan, Perera Rafael (2013). Diagnostic accuracy studies: how to report and analyse inconclusive test results. BMJ Br. Med. J..

[40] Bossuyt Patrick M., Reitsma Johannes B., Bruns David E., Gatsonis Constantine A., Glasziou Paul P., Irwig Les, Lijmer Jeroen G., Moher David, Drummond Rennie, Henrica C W de Vet, Kressel Herbert Y., Rifai Nader, Golub Robert M., Altman Douglas G., Hooft Lotty, Korevaar Daniël A., Cohen Jérémie F., For the STARD group (2015). STARD 2015: an updated list of essential items for reporting diagnostic accuracy studies. Clin. Chem..

[41] Kutlar Abdullah, Kutlar Ferdane, Wilson Jerry B., Headlee Marsha G., Huisman Titus H.J. (1984). Quantitation of hemoglobin components by high-performance cation-exchange liquid chromatography: its use in diagnosis and in the assessment of cellular distribution of hemoglobin variants. Am. J. Hematol..

[42] Ryan K., Bain B.J., Worthington D., James J., Plews D., Mason A., Roper D., Rees D.C., de la Salle B., Streetly A. (2010). Significant haemoglobinopathies: guidelines for screening and diagnosis. Br. J. Haematol..

[43] Keren David F., Hedstrom Deborah, Gulbranson Ronald, Ou Ching-Nan, Bak Richard (2008). Comparison of Sebia capillarys capillary electrophoresis with the primus high-pressure liquid chromatography in the evaluation of hemoglobinopathies. Am. J. Clin. Pathol..

[44] Borbely Nicole, Phelan Lorraine, Szydlo Richard, Bain Barbara (2013). Capillary zone electrophoresis for haemoglobinopathy diagnosis. J. Clin. Pathol..

[45] Nusrat Maliha, Moiz Bushra, Nasir Amna, Rasool Hashmi Mashhooda (2011). An insight into the suspected HbA2' cases detected by high performance liquid chromatography in Pakistan. BMC Res. Notes.

[46] Sharma Prashant, Das Reena (2016). Cation-exchange high-performance liquid chromatography for variant hemoglobins and HbF/A2: what must hematopathologists know about methodology?. World J. Methodol..

[47] Kreuels Benno, Kreuzberg Christina, Kobbe Robin, Ayim-Akonor Matilda, Apiah-Thompson Peter, Thompson Benedicta, Ehmen Christa, Adjei Samuel, Langefeld Iris, Adjei Ohene, May Jürgen (2010). Differing effects of HbS and HbC traits on uncomplicated falciparum malaria, anemia, and child growth. Blood.

[48] Fucharoen Suthat, Weatherall David J. (2012). The hemoglobin E thalassemias. Cold Spring Harbor Perspect. Med..

[49] Warghade Sandeep, Britto Jyothi, Haryan Reshma, Dalvi Tejaswi, Bendre Rajesh, Chheda Pratiksha, Matkar Sunmeet, Salunkhe Yogita, Chanekar Milind, Shah Nilesh (2018). Prevalence of hemoglobin variants and hemoglobinopathies using cation-exchange high-performance liquid chromatography in central reference laboratory of India: a report of 65779 cases. J. Lab. Phys..

[50] Masiello David, Heeney Matthew M., Adewoye Adeboye H., Eung Shawn H., Luo Hong-Yuan, Steinberg Martin H., Chui David.H.K. (2007). Hemoglobin SE disease—a concise review. Am. J. Hematol..

[51] Xu Julia Z., Riolueang Suchada, Glomglao Waraporn, Tachavanich Kalaya, Suksangpleng Thidarat, Ekwattanakit Supachai, Viprakasit Vip (2019). The origin of sickle cell disease in Thailand. Int. J. Lab. Hematol..

[52] Colah Roshan, Gorakshakar Ajit, Nadkarni Anita (2010). Global burden, distribution and prevention of β-thalassemias and hemoglobin E disorders. Expet Rev. Hematol..

