# Supplementary material for: Multispectral imaging for MicroChip electrophoresis enables point-of-care newborn hemoglobin variant screening
Source: Heliyon. 2022 Nov 28;8(12):e11778. doi: 10.1016/j.heliyon.2022.e11778 (PMC9719904; doi:10.1016/j.heliyon.2022.e11778)
Supplement: Supplementary Information.Revision2 [file mmc1.docx]

**Multispectral Imaging for MicroChip Electrophoresis Enables Point-of-Care Newborn Hemoglobin Variant Screening**

Ran An^1,#,*^, Yuning Huang^1,#^, Anne Rocheleau^2,#^, Alireza Avanaki^2^, Priyaleela Thota^2^, Qiaochu Zhang^1^, Yuncheng Man^1^, Zoe Sekyonda^3^, Catherine I. Segbefia^4,5^, Yvonne Dei-Adomakoh^5,6^, Enoch Mensah^6^, Kwaku Ohene-Frempong^7^, Isaac Odame^8,9^, Amma Owusu-Ansah^10^, Umut A. Gurkan^1,3,11,*^

^1^Department of Mechanical and Aerospace Engineering, Case Western Reserve University, Cleveland, OH, USA

^2^HemexHealth, Inc, Portland, OR, USA

^3^Department of Biomedical Engineering, Case Western Reserve University, Cleveland, OH, USA

^4^Department of Child Health, University of Ghana Medical School, Accra, Ghana

^5^Korle Bu Teaching Hospital, Accra, Ghana

^6^Department of Hematology, University of Ghana Medical School, Accra, Ghana

^7^Sickle Cell Foundation of Ghana, Kumasi, Ghana

^8^Division of Hematology/Oncology, The Hospital for Sick Children, Toronto, Ontario, Canada.

^9^Department of Pediatrics, University of Toronto, Toronto, Ontario, Canada.

^10^Department of Pediatrics, Division of Hematology and Oncology, University Hospitals Rainbow Babies and Children’s Hospital, Case Western Reserve University, Cleveland, OH, USA

^11^Case Comprehensive Cancer Center, Case Western Reserve University, Cleveland, OH, USA

# indicates equal contribution

*** Corresponding authors:**

Ran An, PhD

Senior Research Associate

Case Western Reserve University

Office: Glennan 611, 10900 Euclid Ave., Cleveland, OH 44106.

Telephone: +1 (908) 552-9938

E-mail: [rxa407@case.edu](mailto:rxa407@case.edu)

Umut A. Gurkan, PhD

Warren E. Rupp Associate Professor

Case Western Reserve University

Office: Glennan 616B, 10900 Euclid Ave., Cleveland, OH 44106.

Telephone: +1 (216) 368-6447

E-mail: [umut@case.edu](mailto:umut@case.edu)

**
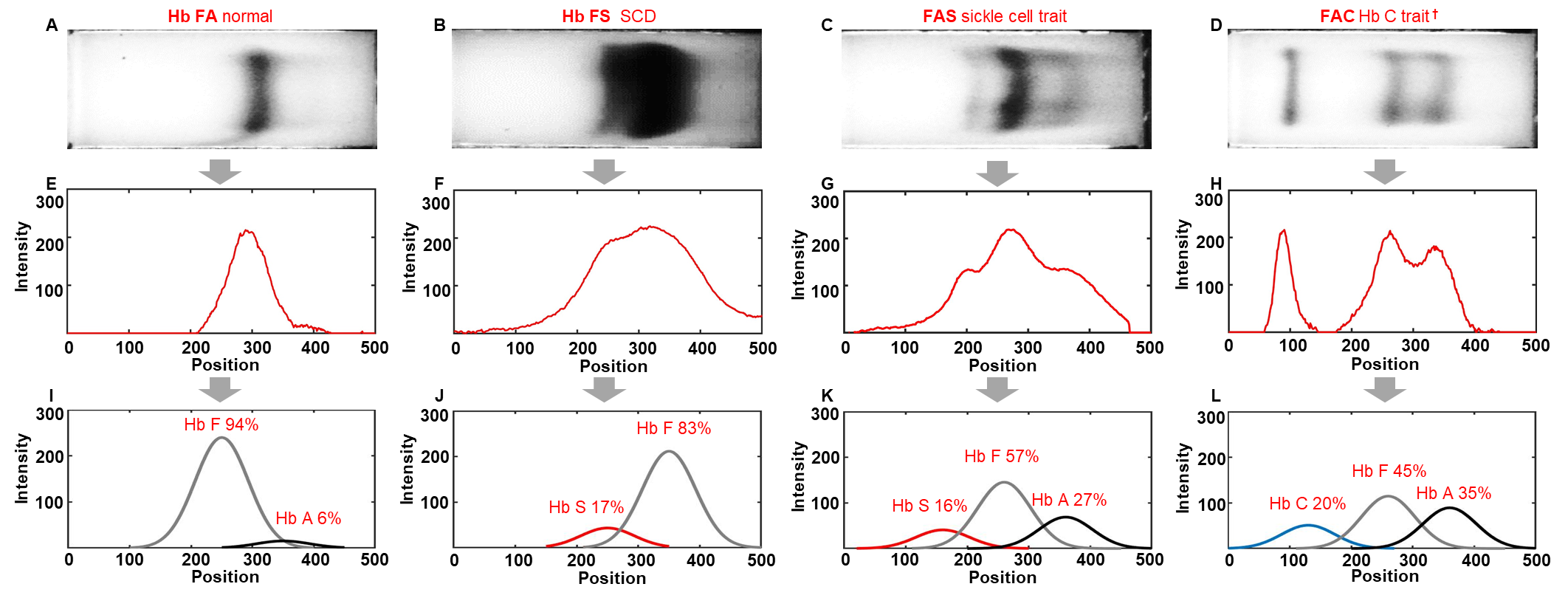
**

**Supplementary Figure 1: Original captured Hb bands under UV field.** (**A-D**) The first row shows images captured under UV light field. **(E-H)** The second row the original captured Hb bands used to regenerate the electropherograms in the third row **(I-L)**.

**
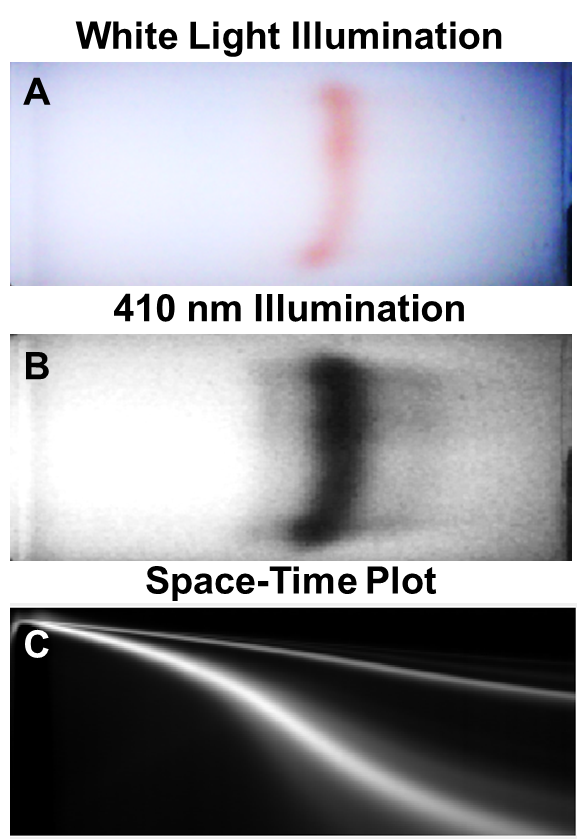
**

**Supplementary Figure 2: Representative test result for a sample with HbS around Gazelle-Multispectral limit of detection. (A)** Image captured under white light illumination. **(B)** Image captured under 410 nm illumination. **(C)** The space-time plot for the presented test. Test result: Gazelle-Multispectral vs. HPLC: HbF = 87% vs. 85%, HbA = 9% vs. 10%, and HbS = 4% vs. 5%.

**Supplementary Table 1:** Number of subjects at specific age

| Age | Number of subjects |
| --- | --- |
| 0 days | 18 |
| 1 day | 130 |
| 2 days | 46 |
| 3 days | 56 |
| 4 days | 2 |
| 5 days | 3 |
| 6 days | 3 |
| 8 days | 2 |
| 9 days | 1 |
| 10 days | 1 |
| 12 days | 1 |
| 14 days | 2 |
| 1 month | 3 |
| 2 months | 50 |
| 3 months | 42 |
| 4 months | 38 |
| 5 months | 25 |
| 6 months | 18 |
| Total | 441 |

**Supplementary Table 2:** Test data quality control results

|  | **0-3 days** | **4-28 days** | **28 days - 6 months** | **Total** |
| --- | --- | --- | --- | --- |
| **Total** | 250 | 15 | 176 | 441 |
| **Valid** | 216 | 11 | 138 | 365 |
| **Inconclusive** | 34 | 4 | 38 | 76 |

**Supplementary Table 3:** Gazelle-Multispectral limit of detection (LOD) test

|  | LOD sample 1 | LOD sample 2 | LOD sample 3 | LOD sample 4 | LOD sample 5*^a^* | LOD sample 6 | LOD sample 7 |
| --- | --- | --- | --- | --- | --- | --- | --- |
| HbS (HPLC) | 11.0% | 7.9% | 7.1% | 4.2% | 3.3% | 2.2% | 0.9% |
| HbF (HPLC) | 73.7% | 76.3% | 76.9% | 79.4% | 80.3% | 81.1% | 82.2% |
| Test 1 | Detected | Detected | Detected | Detected | Detected | Detected | Undetected |
| Test 2 | Detected | Detected | Detected | Detected | Detected | Detected | Undetected |
| Test 3 | Detected | Detected | Detected | Detected | Detected | Undetected | Undetected |

*^a^*The lowest concentration of Hb S levels for which all three replicates scored positive is 3.3%.

**Supplementary Table 4:** Gazelle-Multispectral limit of detection (LOD) test – continued

|  | LOD sample 1 | LOD sample 2 |
| --- | --- | --- |
| HbS (HPLC) | 3.3% | 3.7% |
| HbF (HPLC) | 85.1% | 85.8% |
| Number of Gazelle tests | 10 | 10 |
| Number of tests detected HbS | 10 | 10 |
| Number of tests did not detected HbS | 0 | 0 |

**Supplementary Table 5:** Gazelle-Multispectral screening sensitivity, specificity, positive predictive value (PPV), and negative predictive value (NPV) in comparison to reference standard method – subjects from 28 days to 6 months old*^a^*.

|  | **Disease vs. Normal***^b^* | **Disease vs. Trait***^c^* | | **Trait vs. Normal***^d^* |
| --- | --- | --- | --- | --- |
| **True positive**, TP | 2 | 2 | 31 | |
| **True negative**, TN | 106 | 31 | 106 | |
| **False Positive**, FP | 0 | 0 | 0 | |
| **False negative**, FN | 0 | 0 | 0 | |
| **Sensitivity**, TP/(TP + FN) | 100.0% | 100.0% | 100.0% | |
| **Specificity**, TN/(TN + FP) | 100.0% | 100.0% | 100.0% | |
| **PPV**, TP/(TP + FP) | 100.0% | 100.0% | 100.0% | |
| **NPV**, TN/(TN + FN) | 100.0% | 100.0% | 100.0% | |

*^a^*Out of the 176 tests, 138 tests were recognized as ‘Valid’, 30 tests were recognized as ‘Inconclusive without result’, 4 tests were recognized as ‘Inconclusive – Likely sickle cell trait’, 3 tests were recognized as ‘Inconclusive hemoglobin pattern’, and 1 HPLC result was ‘Inconclusive hemoglobin pattern’

*^b^*SS/SC/FS/FSC vs. AA/FA

*^c^*SS/SC/FS/FSC vs. AS/AC/FAS/FAC

*^d^*AS/AC/FAS/FAC vs. AA/FA
